# Human Organ Tissue Identification by Targeted RNA Deep Sequencing to Aid the Investigation of Traumatic Injury

**DOI:** 10.3390/genes8110319

**Published:** 2017-11-10

**Authors:** Erin Hanson, Jack Ballantyne

**Affiliations:** 1 National Center for Forensic Science, University of Central Florida, Orlando, FL 32816, USA; Erin.Hanson@ucf.edu; 2 Department of Chemistry, University of Central Florida, Orlando, FL 32816, USA

**Keywords:** massively parallel sequencing, human organ tissue, mRNA, tissue identification, forensic science

## Abstract

Molecular analysis of the RNA transcriptome from a putative tissue fragment should permit the assignment of its source to a specific organ, since each will exhibit a unique pattern of gene expression. Determination of the organ source of tissues from crime scenes may aid in shootings and other investigations. We have developed a prototype massively parallel sequencing (MPS) mRNA profiling assay for organ tissue identification that is designed to definitively identify 10 organ/tissue types using a targeted panel of 46 mRNA biomarkers. The identifiable organs and tissues include brain, lung, liver, heart, kidney, intestine, stomach, skeletal muscle, adipose, and trachea. The biomarkers were chosen after iterative specificity testing of numerous candidate genes in various tissue types. The assay is very specific, with little cross-reactivity with non-targeted tissue, and can detect RNA mixtures from different tissues. We also demonstrate the ability of the assay to successful identify the tissue source of origin using a single blind study.

## 1. Introduction

A number of criminal cases requiring forensic investigation involve significant trauma to the human body, in which internal organ tissue is transferred from the injured party to another individual, item, or location [1]. Examples include tissue adhering to bullets that have exited the body, tissue present on the clothing of an individual responsible for causing the trauma through his/her proximity to the victim through the use of a firearm, knife or other implement, tissue present on a suspected murder weapon, and tissue present on the walls, ceilings, or furnishings of the scene of a suspected homicide or serious assault in which the body of a missing person has been removed. The nature of the transferred tissue would be dependent upon the circumstances of the crime, but could include adipose, skeletal muscle, lung, liver, heart, brain, kidney, stomach, and intestine. In combination with standard DNA analysis to identify the individual source of the transferred biological material, the positive identification and differentiation of the organ tissue from blood or other secreted body fluids can provide important probative information.

The identification of traces of desiccated organ tissue can be problematic, and normally requires the expertise of a cellular pathologist and/or histologist, and the use of immunohistochemistry methods [2]. Investigators and/or forensic scientists often have limited, if any, access to such personnel and facilities. In any case, many such tissues are intractable to such analysis due to limited material, and/or the fact that the cellular structures are non-canonical in appearance due to dehydration, and are difficult to discern due to limited quantity or crushing damage. Thus, at present, many case situations involving organ tissue are resolved at the DNA level alone without the investigator being able to ascertain potentially important contextual information about the organ tissue source of the DNA on the person, weapon, or other item.

The goal of the present work was to develop molecular methods that forensic geneticists can use routinely when appropriate to identify internal organ tissue using massively parallel sequencing methodology. The ready availability of molecular methods would supplement or in some cases supplant microscopic methods used by cellular pathologists or histologists to identify the tissue, and allow the positive identification of tissue present in trace amounts and/or sufficiently damaged to preclude standard microscopic identification. A molecular analysis of the RNA transcriptome, the proteome, or the epigenome from a putative tissue fragment should permit assignment of its source to a specific tissue and organ, since each differentiable cell type will exhibit unique patterns of gene and protein expression, as well as DNA methylation [3,4]. These “-omes” are currently the subject of investigation for the purposes of secreted body fluid identification for forensic purposes, and show great promise in that regard [5,6,7,8,9,10,11,12,13,14,15,16,17,18,19,20,21,22,23,24,25,26,27,28,29,30,31,32,33,34,35,36,37]. The authors are unaware of any published or presented work yet on organ tissue identification for forensic purposes using DNA methylation, although this might be expected in the future. A recent publication using mass spectrometry-based proteome analysis reported the identification of specific markers for a limited number of bovine tissues, with the cognate human biomarkers being inferred [38].

Our preferred approach to organ tissue identification, as described in this paper, is based upon an analysis of selected regions of the transcriptome using targeted RNA expression analysis. Terminally differentiated cells in organs and tissues have a unique pattern of gene expression, with approximately 10% of the transcripts being encoded by tissue-enriched genes, with some genes being enriched to such an extent that the mRNA levels in one tissue type are at least five times the maximum levels of all other analyzed tissues [39]. Lindenbergh and colleagues at the Netherlands Forensic Institute (NFI) developed a first-generation capillary electrophoresis (CE)-based mRNA profiling multiplex assay for the inference of the presence of organ tissue in forensic casework [24]. The NFI assay comprises a 17-biomarker set designed to identify six internal organ tissues (brain, lung, liver, skeletal muscle, heart, and kidney). However, since it is a multiplexed CE-based system, the number of incorporated biomarkers per tissue is necessarily limited (two genes per tissue in general, plus housekeeping genes, blood, and skin). Although of great utility, CE-based systems cannot positively associate a tissue marker with a DNA profile in mixed samples [40], unlike sequence-based systems such as massively parallel sequencing (MPS), which could use single nucleotide polymorphisms (SNPs) present in mRNA transcripts (RNA–SNPs) to associate the body fluid specific transcript with one of the admixed DNA profiles. Here, we report the development of a prototype MPS mRNA profiling assay for organ tissue identification designed to definitively identify 10 organ/tissue types using a targeted panel of 46 mRNA biomarkers. The identifiable organs and tissues include brain, lung, liver, heart, kidney, intestine, stomach, skeletal muscle, adipose, and trachea.

## 2. Materials and Methods

### 2.1. Preparation of Body Fluid Stains

Tissue total RNA samples (brain (N = 5), lung (N = 3), liver (N = 4), skeletal muscle (N = 4), heart (N = 4), kidney (N = 3), adipose (N = 2), small intestine (N = 4), stomach (N = 3), trachea (N = 3), colon (N = 1), and spinal cord (N = 1) were purchased from commercial sources (Thermo Fisher Scientific, CA, USA; BioChain^®^, Newark, CA, USA; Clontech, Mountain View, CA, USA; Zyagen, San Diego, CA, USA). All tissue total RNA samples were stored at −40 °C until needed.

Body fluids were collected from volunteers using procedures approved by the University of Central Florida’s Institutional Review Board (SBE-14-10768; approved 11/2014). Informed written consent was obtained from each donor. Blood samples (N = 4) were obtained from commercial sources (Bioreclamation IVT (Long Island, NY, USA), ethlyenediaminetetraacetic acid (EDTA)-containing vacutainers) and 50 μL aliquots were dried onto cotton cloth. Freshly ejaculated liquid semen (N = 4) was provided in sealed plastic tubes and stored frozen until being dried onto sterile cotton swabs (IntegriSwabs, Lynn Peavey, Lenexa, KS, USA). Buccal samples (saliva, N = 4) were collected from donors using sterile cotton swabs by swabbing the inside of the donor’s mouth. Semen-free vaginal secretions (N = 4) and menstrual blood (N = 4) were collected using sterile cotton swabs.

### 2.2. RNA Isolation

Total RNA was extracted from blood, semen, saliva, vaginal secretions, and menstrual blood with guanidine isothiocyanate-phenol:chloroform (Ambion by Thermo Fisher Scientific) and precipitated with isopropanol [18]. Briefly, 500 μL of pre-heated (56 °C for 10 min) denaturing solution (4 M guanidine isothiocyanate, 0.02 M sodium citrate, 0.5% sarkosyl, 0.1 M β-mercaptoethanol) was added to a 1.5 mL Safe Lock extraction tube (Eppendorf, Westbury, NY, USA) containing the stain or swab. The samples were incubated at 56 °C for 30 min. The swab or stain pieces were then placed into a DNA IQ^TM^ spin basket (Promega, Madison, WI, USA), re-inserted back into the original extraction tube, and centrifuged at 14,000 rpm (16,000× *g*) for 5 min. After centrifugation, the basket with swab/stain pieces was discarded. The following was added to each extract: 50 μL 2 M sodium acetate and 600 μL acid phenol:chloroform (5:1), pH 4.5 (Ambion by Thermo Fisher Scientific). The samples were then centrifuged for 20 min at 14,000 rpm (16,000× *g*). The RNA-containing top aqueous layer was transferred to a new 1.5 mL microcentrifuge tube, to which 2 μL of GlycoBlue^TM^ glycogen carrier (Thermo Fisher Scientific) and 500 μL of isopropanol were added. RNA was precipitated for 1 h at −20 °C. The extracts were then centrifuged at 14,000 rpm (16,000× *g*) for 20 min. The supernatant was removed, and the pellet was washed with 900 μL of 75% ethanol/ 25% diethylpyrocarbonate (DEPC)-treated water. Following centrifugation for 10 min at 14,000 rpm (16,000× *g*), the supernatant was removed, and the pellet dried using vacuum centrifugation (56 °C) for 3 min. Twenty microliters of pre-heated (60 °C for 5 min) nuclease-free water (Thermo Fisher Scientific) was added to each sample, followed by incubation at 60 °C for 10 min. Extracts were used immediately or stored at −20 °C until needed.

### 2.3. DNase I Digestion

DNase digestion was performed using the TURBO^TM^ DNA kit (Thermo Fisher Scientific) according to the manufacturer’s protocol. Briefly, 1X TURBO^TM^ DNase Buffer and 1 μL TURBO DNase was added to the 20 μL RNA extracts and incubated at 37 °C for 30 min and 75 °C for 10 min.

### 2.4. RNA Quantification

RNA extracts were quantificated with Quant-iT^TM^ RiboGreen^®^ RNA Kit (Thermo Fisher Scientific) according to the manufacturer’s protocol. Fluorescence was determined using a Synergy^TM^ 2 Multi-Mode microplate reader (BioTek Instruments, Inc., Winooski, VT, USA).

### 2.5. TruSeq^®^ Targeted RNA Library Preparation

MPS libraries of targeted body fluid gene candidates were prepared using the TruSeq^®^ Targeted RNA kit (January 2016 protocol version; Illumina Inc., San Diego, CA, USA) and a TruSeq^®^ Targeted RNA custom oligonucleotide pool (referred to here as TOP) designed using Illumina Design Studio (see Table 1 for final 46-plex assay). All 48- or 96-sample thermal cycler reactions were performed on the Mastercycler^®^ pro S thermal cycler (Eppendorf, Hauppauge, NY, USA) using thin-walled skirted Microseal^®^ Polymerase Chain Reaction (PCR) plates (BIO-RAD, Hercules, CA, USA) sealed with Microseal^®^ B or A (for the amplification reaction) film (BIO-RAD). All 48- or 96-sample purification reactions (requiring the use of magnetic beads) were performed in 0.8 mL 96-well storage plates (Thermo Fisher Scientific) and sealed with Microseal^®^ B film (BIO-RAD) and a magnetic stand-96 (Thermo Fisher Scientific).

RNA was first transcribed into first strand complementary DNA (cDNA) following the TruSeq^®^ Targeted RNA kit intact RNA protocol. The 10 μL reaction consisted of 5 μL of reaction mix: 4 μL reverse transcription cDNA synthesis master mix (RCS1) (Illumina Inc., San Diego, CA, USA), 1 μL ProtoScript^®^ II reverse transcriptase (New England Biolabs Inc., Ipswich, MA, USA), and up to 5 μL of total RNA (target input 50 ng, except for sensitivity studies in which 25, 10, and 5 ng of total RNA was used). The appropriate amount of nuclease-free water (Thermo Fisher Scientific) was added for a total of 5 μL between sample and water for those samples in which less than 5 μL of sample was needed to achieve the target input. For two tissue mixture samples, 25 ng of total RNA from each tissue was used. For three tissue mixtures, 17 ng of total RNA from each tissue was used. Reaction plates were sealed and vortexed at 1600 rpm for 20 s and centrifuged at 280× *g* for 1 min. Reverse transcription was performed as follows: 25 °C for 5 min, 42 °C for 15 min, 95 °C for 10 min, and an infinite hold at 4 °C. The cDNA samples were used immediately or stored at −20 °C overnight (thawed at room temperature before subsequent use).

The custom TOP was next hybridized to the cDNA. The 10 μL hybridization reaction mix consisted of 5 μL TOP (Illumina Inc.) and 5 μL TE buffer pH 8.0 (Thermo Fisher Scientific). Reaction plates were sealed and vortexed at 1600 rpm for 20 s. Following a 1-min incubation at room temperature, 30 μL of OB1 (paramagnetic streptavidin beads, Illumina Inc.) was added to each well. The plate was sealed and vortexed at 1600 rpm for 1 min. The 50 μL hybridization reactions were performed as follows: 70 °C for 5 min, 68 °C for 1 min, 65 °C for 2.5 min, 60 °C for 2.5 min, 55 °C for 4 min, 50 °C for 4 min, 45 °C for 4 min, 40 °C for 4 min, 35 °C for 4 min, 30 °C for 4 min, and a hold at 30 °C. The bound oligos were then washed, extended, and ligated according to the manufacturer’s protocol (TruSeq^®^ Targeted RNA, January 2016 protocol version; Illumina Inc.). The extension–ligation products were then amplified, and Index 1 (i7) adapters and Index 2 (i5) adapters were added in the process. Each sample received a unique combination of i7 and i5 adapters to permit the pooling of finished libraries prior to sequencing. Twenty microliters of the purified extension–ligation products were used in the 50 μL amplification reaction. The reaction plate was sealed, vortexed at 1600 rpm for 30 s, and centrifuged at 280× *g* for 1 min. The amplification reaction was performed as follows: 95 °C for 2 min, 34 cycles of 98 °C for 30 s, 62 °C for 30 s, 72 °C for 60 s, 72 °C for 5 min, and an infinite hold at 10 °C. Amplification products were used immediately or stored at 4 °C overnight if needed. The individual sample libraries were next purified according to the manufacturer’s protocol (TruSeq^®^ Targeted RNA, January 2016 protocol version; Illumina Inc.), resulting in a final sample library volume of 12.5 μL. Five microliters of each sample library were combined into a single pooled library per sequencing reaction. Pooled libraries and remaining individual libraries were stored at -20 °C until needed.

### 2.6. TruSeq^®^ Targeted RNA Library Quantification

Pooled libraries were quantificated using the 2200 TapeStation (Agilent Technologies, Santa Clara, CA, USA) and High Sensitivity D1000 Screen tape according to the manufacturer’s protocol. Neat and 1:10 diluted libraries were run, and the average concentration obtained from the 100–300 bp region was used to determine the library concentration (in nM).

### 2.7. MiSeq^®^ Sequencing

Pooled libraries were diluted to 4 nM and denatured according to the manufacturer’s recommended protocol. Briefly, 5 μL of the 4 nM library was mixed with 5 μL 0.2 N NaOH and incubated at room temperature for 5 min. To the 10 μL denatured library sample, 990 μL of pre-chilled HT1 buffer (Illumina Inc.) was added, resulting in a 20 pM sample. A 600 μL 6 pM sample was then prepared by further diluting the 20 pM library (180 μL 20 pM denatured sample and 420 μL pre-chilled HT1). The 600 μL 6 pM sample was immediately pipetted into the MiSeq^®^ v3 150 cycle reagent cartridge for sequencing on the MiSeq^®^ instrument (Illumina Inc.) using a v3 flow cell. The sequencing runs consisted of 51 single-end sequencing cycles.

### 2.8. Data Analysis

After sequencing, local sequencing software on the MiSeq analyzed the data (base calling, demultiplexing, and alignment to the provided manifest file using a banded Smith Waterman algorithm), resulting in a target hits file that displays total reads per amplicon per sample. A minimum sample total read count (MTR) of 5000 was used as an individual sample threshold, and samples below the MTR were excluded from analysis. In addition, a minimum biomarker read count (MBR) count of 500 was used as an individual biomarker threshold, with any counts below this threshold removed. A third threshold was then used in which individual biomarker read count values that were less than 0.5% of the total reads for the sample were also removed.

After filtering of samples in accordance with the above thresholds, the raw total read count data was plotted in Microsoft^®^ Excel (Office 2016, Microsoft, Redmond, WA, USA) in order to view the total raw counts per sample, and bar graphs were created to evaluate raw counts by sample and by gene. The percent contribution of total reads (biomarker read count/total count for sample) was determined for each biomarker. The percent contribution of reads was next calculated to provide the percentage of total reads for each individual sample that was attributable to the various tissue- or body fluid-specific markers, and displayed as stacked bar graphs.

Agglomerative hierarchical clustering analysis is an alternative complementary method for data analysis that employs the raw hit counts as input without the use of the ad hoc thresholds described above [41]. Clustering was performed using the BaseSpace^®^ TruSeq^®^ Targeted RNA v1.0 app (Illumina Inc.), which jointly clusters samples and biomarker amplicons. Briefly, the software uses a minimum count threshold of 1, log transforms the counts, and performs median normalization across all the samples. After clustering, the data are median absolute deviation (MAD)-normalized so that the expression values for each gene are on the same scale. Biomarker amplicon, sample dendrogram files, and a clustering heat map are used to visualize the similarities and differences in biomarker expression between samples.

## 3. Results

### 3.1. Assay Development

#### 3.1.1. Candidate Selection

Putative tissue-specific genes were identified through literature and database searches using a priori knowledge of the physiology and biochemistry of the tissue of interest. Additionally, attempts were made to include the tissue-specific biomarkers from Lindenbergh et al. [24] for consistency in forensic tissue identification assays for the six tissues (brain, lung, liver, skeletal muscle, heart, and kidney) in common between the two assays. Of the 13 candidates from the Lindenbergh assay [24], all genes were evaluated for use in the targeted RNA sequencing assay except the kidney biomarker FXYD2 (FXYD domain containing ion transport regulator 2), as no commercial off-the-shelf assay was readily available for this biomarker. Of the 12 biomarkers tested, nine biomarkers were included: brain–SNAP25 (synaptosomal-associated protein 25), RTN1 (reticulon-1); lung—SFTPB (surfactant protein B), SFTPD (surfactant protein D); liver—AMBP (alpha-1-microglobulin/bikunin precursor); skeletal muscle—TNNI2 (troponin I2); heart—MYBPC3 (myosin binding protein C); heart muscle—ITGB1BP3 (integrin beta 1 binding protein 3, or NMRK2 (nicotinmide riboside kinase 2), which was identified as a general muscle candidate by Lindenbergh et al., but demonstrated heart muscle specificity in the current assay); kidney–UMOD (uromodulin). The targeted RNA sequencing assay sought to include biomarkers for several additional tissues, including trachea, adipose, intestine, and stomach. We designed and evaluated three targeted oligonucleotide primer pools (TOPs; TOP1—64-plex; TOP2—48-plex; and TOP3—46-plex), which resulted in the evaluation of a total of 77 gene candidates for appropriate specificity (brain—nine candidates; lung—eight candidates; trachea—one candidate; liver—nine candidates; skeletal muscle—14 candidates; heart—seven candidates; kidney—eight candidates; adipose—seven candidates; intestine—nine candidates; stomach—five candidates).

**Figure 1:**
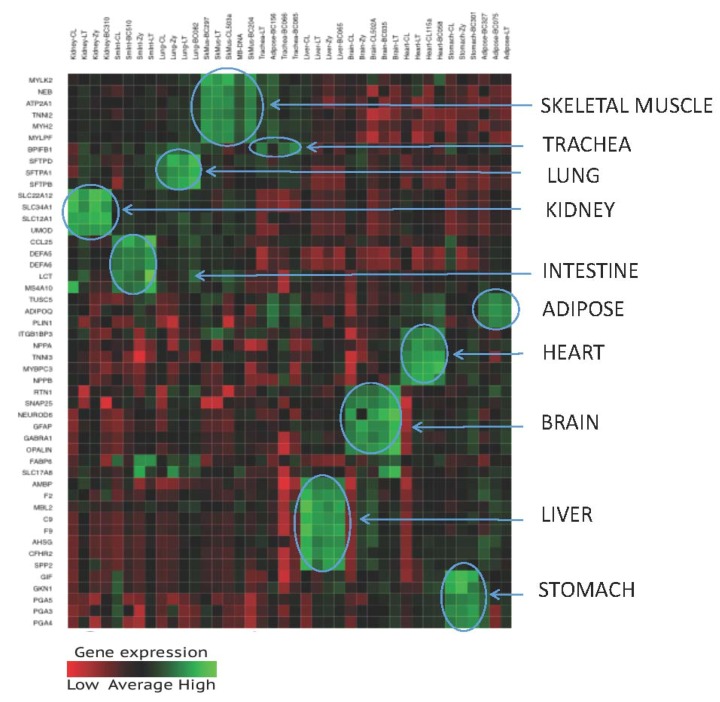
Gene Expression Heat Map of 48 Tissue-Specific Markers in 10 Tissues (Skeletal muscle, Trachea, Lung, Kidney, Intestine, Heart, Adipose, Brain, Liver, and Stomach). Y-axis—biomarkers (genes); X-axis—tissue samples. Green represents higher expression, red represents lower expression. Clusters of up-regulated gene expression of a group of biomarkers specific to the target tissue are highlighted with blue circles.

Individual gene candidates were evaluated for specificity (e.g., ideal candidates with high read counts in target tissues and low or no read counts in non-target tissues, and other forensically relevant biological fluids (blood, semen, saliva, vaginal secretions, and menstrual blood)) and abundance (e.g., ideal candidates with consistently moderate to high read counts amongst different donors of the target tissue). Expression heat maps (Figure 1) were generated for each TOP design after initial testing. The heat maps provided easy visualization of gene expression to select suitable candidates. Numerous gene candidates were not selected for use in the targeted RNA sequencing assay due to various factors such as poor performance (amplification efficiency), low abundance, or cross-reactivity with non-target tissues (Table S1).

The iterative selection process resulted in the development of a final 46-plex assay (Table 1) that was determined to be suitable for further testing and evaluation. This assay contained six brain biomarkers, three lung biomarkers, one trachea biomarker, eight liver biomarkers, six skeletal muscle biomarkers, five heart biomarkers (with one potentially more specificity to heart muscle), four kidney biomarkers, three adipose biomarkers, five intestine biomarkers, and five stomach biomarkers.

#### 3.1.2. Specificity of the 46—Plex Targeted RNA Sequencing Assay

The initial performance of the 46-plex tissue identification assay was evaluated in 35 total RNA samples (brain (N = 5), lung (N = 3), trachea (N = 3), liver (N = 4), skeletal muscle (N = 4), heart (N = 4), kidney (N = 3), adipose (N = 2), small intestine (N = 4), and stomach (N = 3)) obtained from commercial sources. Fifty ng of input total RNA was used for all samples. Raw read count data was evaluated using previously developed ad hoc thresholds for RNA MPS data analysis: (1) MTR of 5000 for individual samples, and (2) minimum MBR of 500 and a minimum 0.5% total read count threshold for individual biomarkers. Samples with total read counts below the MTR (5000) were not analyzed. Read counts that were below the MBR and that did not represent at least 0.5% of the total read counts for the sample were removed (read counts converted to 0). All of the 35 tissue samples well exceeded the MTR, and were therefore all included in the analysis.

The read count values for each biomarker were averaged for each tissue type amongst the 35 samples to evaluate the specificity of the included biomarkers (Table 2). It is important to note that the read count values used in the averages are not normalized to total sample counts. Therefore, the observed variation in read counts between samples will be quite large. This does not negatively affect interpretation of the tissue source, as other analysis metrics will be described in subsequent sections using normalized data.

Amongst the five brain donors tested, the average read count for brain biomarkers ranged from 167,707 (SNAP25, 1 standard deviation (S.D.) = 47,884) to 9698 (OPALIN (oligodendrocytic myelin paranodal and inner loop protein), 1 S.D. = 5125). Expression was not observed for all of the other non-brain biomarkers, except for liver biomarkers AMBP and AHSG (alpha 2-HS glycoprotein. Expression of these biomarkers was only observed in one of the five donors, and therefore was not reproducible. Therefore, it is not likely that these biomarkers will confound the ability to definitively identify brain tissue.

For lung, amongst the three donors tested, the average read count for lung biomarkers ranged from 145,365 (SFTPB, 1 S.D. = 97,488) to 56,487 (SFTPD, 1 S.D. = 51,113). Expression of the trachea biomarker BPIFB1 (BPI fold containing family B member 1) was present in two of the three lung samples tested, with expression levels only ~8% that of the highest expressing lung biomarker SFTPB and ~6% that of BPIFB1 in trachea samples. Additionally, lung and trachea are connected tissue, and the lung samples may contain small amounts of trachea tissue. Expression was not observed for any other non-lung biomarker with the exception of RTN1 (brain), in which a low read count (3784) was observed in only one of the three samples tested. This latter level of expression should not have any impact on the ability to identify lung tissue.

For trachea, BPIFB1 was detected with high abundance in the three trachea samples tested, with a total read count of 190,738 (1 S.D. = 135,931). Low expression levels of several non-trachea genes were observed in the trachea samples, with the most substantial expression observed for heart candidate NPPA (natriuretic peptide A), with a total read count of 7182. This NPPA expression level was only observed in one of the three trachea samples, and therefore was not reproducible amongst the small sample set tested. The low-level expression in trachea from a small number of ‘non-trachea’ biomarkers were not reproducible across all trachea samples examined, with the exception of RTN1 (brain biomarker) and PLIN1 (perilipin 1, adipose biomarker). Future work will seek to identify additional specific trachea markers. Trachea was not originally one of the target tissues intended for the assay, with BPIFB1 originally identified as a possible lung biomarker. However, after initial testing, it was evident that it demonstrated specificity for trachea tissue, and therefore the assay was expanded to include trachea as a target tissue.

For liver, amongst the four donors tested, the average read count for liver biomarkers ranged from 165,375 (AMBP, 1 S.D. = 31,427) to 4649 (MBL2 (mannose binding lectin 2), 1 S.D. = 2320). Expression was not observed for any of the other non-liver biomarkers. Expression of MBL2 and SPP2 (secreted phosphoprotein 2) was observed in only three of the four liver samples. Due to the lower expression of these biomarkers and the reasonable number of liver biomarkers exhibiting moderate to high expression, MBL2 and SPP2 will likely be removed in subsequent assay iterations.

For skeletal muscle, amongst the four donors tested, the average read count for skeletal muscle biomarkers ranged from 125,702 (MYLPF (myosin light chain, phosphorylatable, fast skeletal muscle), 1 S.D. = 48,208) to 19,082 (MYLK2 (myosin light chain kinase 2), 1 S.D. = 11,341). Expression was not observed for any of the other non-skeletal muscle biomarkers, with the exception of ITGB1BP3 (heart muscle), which was detected in only one of the four skeletal muscle samples at a low expression level (2403), and therefore not reproducible.

For heart, amongst the four donors tested, the average read count for heart biomarkers ranged from 168,146 (TNNI3 (troponin I3), 1 S.D. = 27,231) to 14,539 (NPPB (natriuretic peptide B), 1 S.D. = 12,336). Expression was not observed for any of the other non-heart biomarkers, with the exception of PLIN1 (adipose biomarker), which was detected in only one of the four heart samples at a low expression level (2251). Expression of this biomarker in heart samples was therefore not reproducible (present in only one donor), and should have no impact on the ability to definitively identify lung tissue.

For kidney, amongst the three donors tested, the average read count for kidney biomarkers ranged from 53,914 (UMOD, 1 S.D. = 32,933) to 9133 (SLC22A12 (solute carrier family 22 member 12), 1 S.D. = 5206). Expression was not observed for any of the other non-kidney biomarkers.

For adipose, amongst the two donors tested, the average read count for adipose biomarkers ranged from 21,176 (PLIN1, 1 S.D. = 10,898) to 6842 (TUSC5 (tumor suppressor candidate 5), 1 S.D. = 366). Adipose was the most challenging tissue to identify during the development of this assay. While the adipose biomarkers demonstrated a high degree of specificity for adipose tissue (i.e., no substantial expression in other tissue types), the expression of skeletal muscle, stomach, and liver biomarkers was observed in one of the two samples. The expression profiles for the two adipose samples were slightly different, with expression of the skeletal muscle biomarkers observed in one of the samples, and expression of the liver and stomach biomarkers in the other sample. The anatomical location from which the adipose tissue samples were taken was not known, but could account for these differences in expression profiles. Further work with additional adipose samples is needed to better determine the extent to which the anatomical location affects the combinatorial expression signatures of included biomarkers. The expression observed in the skeletal muscle biomarkers is relatively low, with more substantial expression observed for the stomach biomarkers. Despite these challenges, adipose tissue was identifiable throughout the study due to its unique expression pattern (e.g., adipose–muscle–stomach biomarkers). The expression level of the skeletal muscle biomarkers was 17–40% higher in skeletal muscle tissue compared with adipose tissue. The expression of the stomach biomarkers PGA3 (pepsinogen 3, group 1) and PGA4 (pepsinogen 4, group 1) was only 11–13% higher, respectively, in stomach tissue. Expression levels similar to that of stomach tissue were observed for PGA5 (pepsinogen 5, group 1), although expression was found only in one of the two adipose samples tested.

For small intestine, amongst the four donors tested, the average read count for intestine biomarkers ranged from 165,872 (DEFA5 (defensin alpha 5), 1 S.D. = 61,087) to 4333 (CCL25 (C-C motif chemokine ligand 25), 1 S.D. = 2896). Low expression was observed for stomach biomarkers PGA5, PGA3, and PGA4, again demonstrating the future possibility of removing and replacing these biomarkers from the current assay. LCT (lactase) was only detected in one of the four samples, and CCL25 in only three of the four samples. The low read counts suggest that these biomarkers may be low abundance biomarkers, although both still demonstrated a high degree of specificity for intestine tissue.

For stomach, amongst the three donors tested, the average read count for stomach biomarkers ranged from 155,582 (PGA4, 1 S.D. = 47,938) to 7311 (GIF (gastric intrinsic factor), 1 S.D. = 960). Two of the lower expressing stomach biomarkers, GIF and GKN1 (gastrokine 1), were nevertheless highly specific to their target tissue.

**Figure 2:**
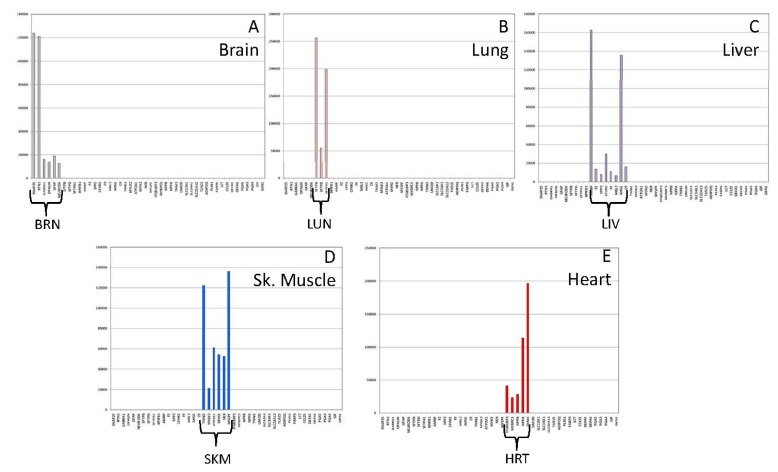
Gene Expression Profiles for Different Individual Tissue Types Using the 46-plex Targeted RNA Sequencing Assay. Read counts for 46 tissue specific genes are shown for individual tissue samples (**A**) brain; (**B**) lung, (**C**) liver, (**D**) skeletal muscle, (**E**) heart. Colored bars represent expression of tissue-specific biomarkers within the target tissues (grey—brain, pink—lung, purple—liver, blue—skeletal muscle, red—heart). Y-axis—read counts, X-axis—tissue-specific genes (order of markers left to right is the same as shown in Table 1 from top to bottom).

**Figure 3:**
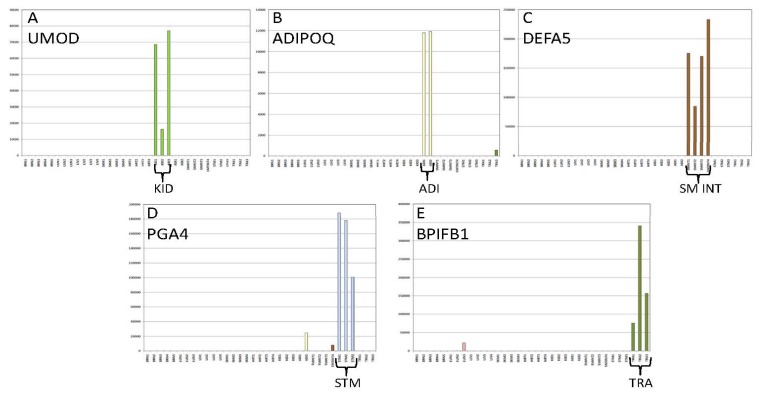
Tissue-Specific Gene Expression Exemplified by Individual Gene Candidates amongst 35 Tissue Samples. Read counts for individual biomarkers (**A**) UMOD (uromodulin), kidney specific; (**B**) ADIPOQ, adipose specific; (**C**) DEFA5, intestine specific; (**D**) PGA4, stomach specific; (**E**) BPIFB1, trachea specific, are shown amongst a set of 35 tissue samples (Brain (BRN), N = 5), lung (LUN, N = 3), liver (LIV, N = 4), skeletal Muscle (SKMUS, N = 4), heart (HRT, N = 4), kidney (KID, N = 3), adipose (ADI, N = 2), small intestine (SMINT, N = 4), stomach (STM, N = 3), trachea (TRA, N = 3). Colored bars represent biomarker expression (i.e., read counts) in the target tissue: light green—kidney, yellow—adipose, brown—intestine, blue—stomach, dark green—trachea). Y-axis—read counts, X-axis—tissue samples.

The threshold-filtered read count data was also visualized with the use of simple bar graphs that were constructed either ‘by sample type’ (Figure 2) or ‘by gene biomarker’ (Figure 3). Figure 2 shows expression data from a single brain (A), lung (B), liver (C), skeletal muscle (D), and heart (E) tissue sample amongst the 46 included biomarkers. As can be seen from these graphs, highly specific expression patterns were observed for the tissues. Figure 3 shows the expression data from 35 tissue samples graphed ‘by gene’, with one representative gene selected for kidney (A, UMOD), adipose (B, ADIPOQ (adiponectin, C1Q and collagen domain containing), intestine (C, DEFA5), stomach (D, PGA4), and trachea (E, BPIFB1). The target specific biomarkers are highly expressed in their respective target tissues.

#### 3.1.3. Tissue Inference

In order to infer the presence of a particular tissue based on quantitative gene expression from the targeted RNA sequencing assay, we have investigated the use of ad hoc binary approaches to tissue prediction. The output from these approaches is a simple categorical statement of the presence or absence of a particular tissue. The two complementary approaches include (1) assigning the percentage of reads in a sample that are due to each of the 10 tissue-specific biomarker classes included in the assay, and (2) the inter-sample differential gene expression revealed by agglomerative hierarchical clustering.

In order to generate tissue-specific read percentages, threshold-filtered read counts for individual biomarkers were divided by the total reads for the sample. The sum of the individual biomarker percentages in the sample comprising each tissue class were totaled to provide the total percentage of reads attributable in the sample to the different tissue classes. The average percent contribution of each biomarker class, as well as the range of percentages observed in the different tissue samples, is shown in Table 3, further demonstrating the high degree of specificity of the included biomarkers for all tissues, with the exception of adipose. For most of tissues, the percentage of reads attributable to their respective tissue-specific biomarkers ranged from 90–100%. A slightly larger range was observed amongst trachea samples, with the percent composition attributable to the trachea biomarker ranging from 79–98%. As described above, the most challenging tissue type was adipose, with the adipose biomarker class found to comprise 25–59% of the biomarkers present in adipose tissue. The lower proportion of the expected biomarker class was primarily due to the co-expression of skeletal muscle and stomach biomarkers in adipose tissue samples. The unique expression profile for adipose tissue (expression from skeletal muscle, adipose, and stomach biomarkers) is clearly discerned based on the percent composition values. As stated previously, adipose tissue was identifiable in these initial studies using only single source tissue samples based on this unique expression profile.

The use of the sample percent composition of each biomarker class expression to identify the presence of a particular tissue or tissues is useful, because it takes into account variability in read counts between library preparations and sequencing runs. It also is not affected by a possible absence of some of the lower expressing biomarkers in a particular sample. The specific biomarker composition within the target specific biomarker classes is interesting to evaluate, as some samples from the same tissue type show very similar biomarker expression profiles, and some show greater variation. Examples of the percent composition from the individual brain and skeletal muscle biomarkers are shown in Figure S1. For the skeletal muscle samples (99–100% of total reads attributable to skeletal muscle class biomarkers), similar expression levels of the individual biomarkers were observed between samples (Figure S1B). The overall percent composition attributable to brain biomarkers was 96–100% amongst the five brain samples tested. The highest contribution for each sample was from SNAP25 (41–61%) followed by RTN1 (11–39%). Interestingly, GFAP (glial fibrillary acidic protein) represented 6–15% of the brain biomarker composition in four of the five samples, but accounted for 38% of brain biomarker composition in the fifth brain sample. This could indicate the potential to identify different anatomical regions within some of the tissue or organ types, particularly the brain. It is possible that the fifth sample was taken from a different region of the brain than the other four samples, and hence GFAP showed higher expression levels in this region.

**Figure 4:**
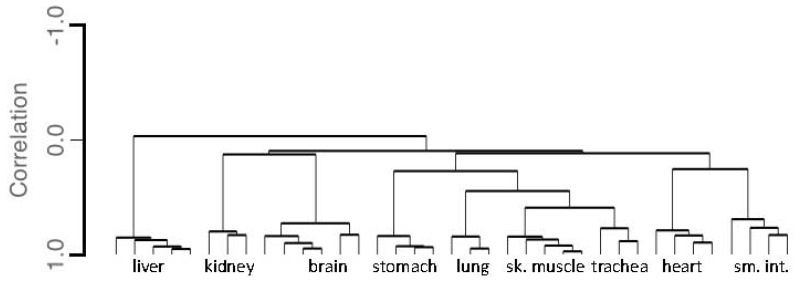
Dendrogram of Single Source Tissue Samples Clustering According to Similarities in Gene Expression. The gene expression correlation distance between samples is indicated by the length of the vertical branch points on the Y-axis.

Evaluation of the similarities and differences in gene expression of the 46 targeted genes between the tissue samples was performed using agglomerative hierarchical clustering analysis. The clustering was performed jointly on samples and biomarker amplicons using unfiltered raw read counts. Results further demonstrate the high degree of specificity of the 46-plex assay with samples of the same tissue type clustering together due to similarities in gene expression. Figure 4 shows a representative dendrogram of the unbiased clustering of tissue samples when samples from liver (N = 5), kidney (N = 3), brain (N = 6), stomach (N = 4), lung (N = 3), trachea (N = 3), skeletal muscle (N = 5), heart (N = 4), and small intestine (N = 4) were analyzed. Adipose was not included for clarity, since additional work is needed to improve the identification of adipose tissue. As can be seen from the clusters the nine different tissue classes show distinct intra-class differences in gene expression, whereas samples of the sample tissue type cluster together.

### 3.2. Performance Testing

We have carried out an initial set of performance checks on the prototype 46-plex assay to determine its potential efficacy for future use in forensic casework and to identify opportunities for improvement.

#### 3.2.1. Biomarker Sensitivity of Detection

The current optimal input for the 46-plex assay is 50 ng of total RNA. However, the sensitivity of detection of numerous biomarkers included in the assay is likely below 50 ng. To evaluate the differing sensitivity of the included biomarkers, we analyzed 25, 10, and 5 ng total RNA inputs for brain, lung, trachea, liver, skeletal muscle, heart, kidney, small intestine, and stomach (N = 1 for each tissue). The results for the sensitivity study are provided in Table 4. The total read counts for each sample, the percent contribution to the sample of the tissue class-specific biomarkers, and the read counts for the individual tissue specific biomarkers within their target tissue are shown. For all of the tissues, with the exception of skeletal muscle, the total sample read count decreased as expected as the input amount of RNA decreased. Almost all of the 5 ng samples (with the exception of trachea and kidney) were still above the MTR. The percent contribution for each tissue attributable to the tissue specific biomarkers associated with each tissue ranged from 92–100% for all samples.

The results provide an indication of the current sensitivity of detection levels of the included biomarkers, and do not necessarily provide an accurate estimate of the limit of detection (LOD) of the assay. The 50 ng input remains a reasonable attainable target for most samples, but may be reduced as additional optimization work is performed.

#### 3.2.2. Mixtures

**Figure 5:**
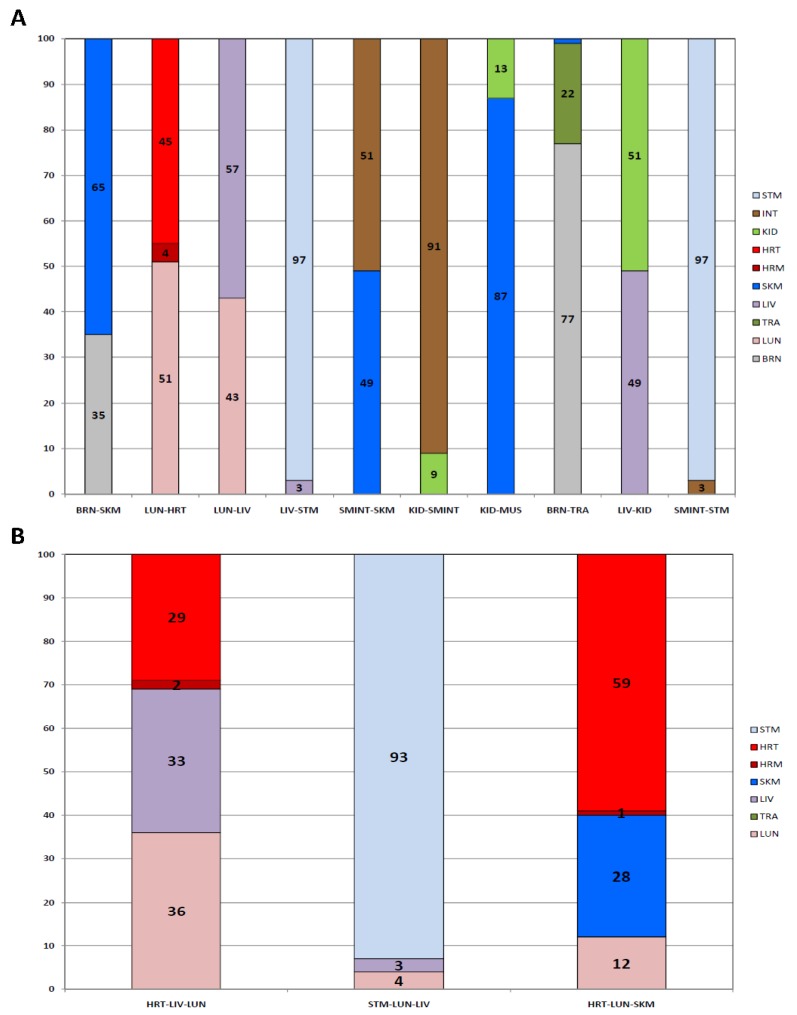
Identified Biomarker Expression Classes in Two- and Three-Tissue Admixed Samples. The percent contributions for individual biomarkers were calculated (reads per biomarker/total reads per sample). The percentages from each group of tissue-specific biomarkers were combined to determine the percentage of reads per sample attributable to each tissue class. Percent reads attributable to each biomarker group are listed and represented by color: grey—brain, pink—lung, dark green—trachea, purple—liver, blue—skeletal muscle, dark red—heart muscle, red—heart, light green—kidney, brown—intestine, blue—stomach. (**A**) binary tissue mixtures, (**B**) ternary tissue mixtures. Brain (BRN), lung (LUN), trachea (TRA), liver (LIV), skeletal Muscle (SKM and MUS), heart muscle (HRM), heart (HRT), kidney (KID), intestine (INT), stomach (STM), small intestine (SMTINT).

In criminal cases undergoing forensic investigation, more than one tissue type may be present in a sample. For example, a bullet recovered after having struck an individual may have traversed through multiple organ tissues. Positive identification and differentiation of the organ tissue(s) present in a sample could provide important probative information. Therefore, we evaluated the performance of the developed 46-plex assay with binary and ternary organ tissue admixtures (Figure 5).

Ten binary mixtures were tested that consisted of the following: brain—skeletal muscle, lung—heart, lung—liver, liver—stomach, small intestine—skeletal muscle, kidney—small intestine, kidney—skeletal muscle, brain—trachea, liver—kidney, and small intestine—stomach. The binary mixtures comprised 25 ng of total RNA from both tissues. The percent composition attributable to each tissue biomarker class is shown for each mixture in Figure 5A. Of the 10 binary mixtures, six showed a substantial contribution from each tissue: brain—skeletal muscle (35%, 65%, respectively); lung—heart (51%, 49%, respectively); lung—liver (43%, 57%, respectively); small intestine—skeletal muscle (51%, 49%, respectively); brain—trachea (77%, 22%, respectively); and liver—kidney (49%, 51%, respectively). The relative proportions of the components were such that identification of the mixture constituents would be a facile matter if such mixtures were to be encountered in a real-life case scenario. For the four remaining mixtures (liver—stomach, kidney—small intestine, kidney—skeletal muscle, and small intestine—stomach), one of the constituents was present as a minor component comprising 3–13% of the total reads. This is likely due to the higher abundance of some biomarker tissue classes compared with others, and such low level relative expression could confound tissue inference of the minor component.

Three ternary mixtures were tested that consisted of the following: heart–liver–lung, stomach–lung–liver, and heart–lung–skeletal muscle. The ternary mixtures comprised 17 ng of total RNA from all three tissues. The percent contributions attributable to each tissue biomarker class is shown for each mixture in Figure 5B. For the heart—liver—lung admixture, each tissue component was successfully detected with almost equal one-third contributions from each of the three tissue biomarker classes. For the heart—lung—skeletal muscle admixture, all three tissues were also identified, with 60% of the total reads attributable to heart biomarkers, 28% to skeletal muscle biomarkers, and 12% to lung biomarkers. The most challenging ternary sample for potential identification purposes was the stomach—lung—liver mixture, in which the percent contribution from stomach biomarkers dominated the sample with 93% of the total reads. Liver and lung were present, but making up only 3% and 4% of the total reads, respectively, which is close to the background transcription noise threshold.

Due to the low-level expression of non-target biomarkers in some tissues, minimum percent contribution thresholds will also need to be established for accurate tissue identification inference. Additional mixture studies will also need to be performed to evaluate observed percent contributions from minor mixture components to assess how those thresholds affect mixture interpretation. From the relative expression data of different biomarker classes in single source samples (Table 3), except for adipose, low-level expression from non-target biomarker classes for the assay range from 1–11%, with trachea showing 11% of reads from non-trachea biomarkers. We expect this value to decrease as additional trachea biomarkers are added to the assay. However, the data provides an initial indication of the magnitude of possible threshold levels for discarding background transcriptional noise that will need to be incorporated into a finalized assay for routine forensic use.

#### 3.2.3. Repeatability

Expression data for the same single source tissue samples (N = 1 for brain, lung, trachea, liver, skeletal muscle, kidney, adipose, small intestine, and stomach) were available from two different sequencing runs (using the same operator and instrument), and therefore the repeatability of the developed assay was evaluated. Figure S2 provides a graphical representation of the percent contributions of the biomarkers in each tissue class for the data from both runs (“-1” and “-2”). The data clearly shows good repeatability of the targeted RNA assay across different sequencing runs, especially considering that the samples were processed via separate library preparations and sequencing runs. Overall, the total read count for each individual sample was ~4–5 times higher in run 1. The -1 samples were from a 48-sample run consisting of more low-input (sensitivity) samples or negative (body fluid) samples. Therefore, more of the read ‘real estate’ was available for positive samples in this run. The -2 samples were from a 96-sample run consisting of mostly ‘positive’ single source or admixed tissue samples. This demonstrates the utility of using the percent composition analysis approach, as it normalizes the values to total read counts, and therefore accounts for any potential run-to-run variation in the total read counts.

#### 3.2.4. Specificity

In addition to the tissue samples, the expression of each of the included biomarkers was evaluated in non-organ tissue body fluid samples commonly found at crime scenes, including blood (N = 5), semen (N = 4), saliva (N = 4), vaginal secretions (N = 4), and menstrual blood (N = 4) (Table S2). Amongst these samples, one blood sample gave a total read count above the MTR. The other four blood samples and all semen, saliva, vaginal secretions, and menstrual samples were below the MTR, indicating no substantial cross-reactivity with forensically relevant body fluids. For the one blood sample above the MTR, read counts were detected only for the brain biomarkers SNAP25 (16,743 counts), RTN1 (5723), and GFAP (1035). In comparison, the average read counts for these biomarkers in brain tissue are 10-, 14-, and 52-fold higher than those observed in the blood sample.

We also tested spinal cord and colon samples (N = 1 each), since they represented tissues with related physiological functions to the included brain and intestine target tissues. The expression results for these samples are provided in Table S2. For spinal cord, substantial expression of brain biomarkers SNAP25, RTN1, and GFAP were observed with little to no expression of GABRA1 (gamma-aminobutyric acid (GABA) A receptor) and NEUROD6 (neuronal differentiation 6). Additional spinal cord samples need to be tested to determine if these three brain genes will be consistently expressed (and the other two brain ones not). For colon, expression of four (LCT, CCL25, DEFA5 and DEFA6 (defensing alpha 6)) of the five intestine biomarkers was observed. Expression of the brain- and intestine-specific biomarkers in the related spinal cord and colon tissues is not entirely unexpected. Attempts will be made in future iterations of the assay to try and distinguish related tissues with additional markers, although a combined intestine/colon or brain/spinal cord designation would also suffice for the assay’s purpose.

#### 3.2.5. DNA and Amplification Blanks

The RNA sample preparation process used for this study includes DNase treatment of all total RNA extracts to remove any residual DNA that may be present in the sample. However, to confirm that DNA would not produce any amplification products that could confound RNA biomarker analysis and interpretation, genomic DNA was tested with the 46-plex assay. Five ng of genomic DNA isolated (from a menstrual blood sample) was run once during two different sequencing runs. In each instance, the total read counts for the DNA sample was below the MTR (0 and 522 total read counts), and confirmed the absence of potentially confounding DNA products. An amplification blank (i.e., nuclease-free water in place of sample) was also included in two different sequencing runs. The total read counts for both amplification blanks was also below the MTR, and therefore was not included in the downstream analysis.

### 3.3. Blind Study

A set of six samples were prepared by analyst 2 and provided to analyst 1 to analyze as a single blind study. Analyst 1 had no knowledge of the sample type prior to conducting the analysis. The samples used as unknowns for the blind study were from individuals who had not been previously run with the 46-plex assay. The raw read count data was threshold-filtered, and the percent contributions from each tissue class were determined for each unknown sample. The percent contributions of biomarker tissue classes for each of the unknown samples as determined by the blinded analyst 1, the true source of the provided samples, and the blinded analyst’s conclusions are provided in Table 5.

Unknown samples 1, 2, 4, 5, and 6 were all above the MTR. Unknown sample 3 was below MTR. Therefore, it was concluded that no organ tissue was detected in this sample, which was a correct assessment, since the sample was a blank (water). Unknown sample 1 had 100% of reads attributable to stomach biomarkers, and was correctly identified as a stomach sample. Unknown sample 2 had 100% of reads attributable to brain, and was correctly identified as a brain sample. The brain sample used was a poly-A enriched brain sample, which is different than the other brain samples used in the study, but this did not preclude facile inference of brain. Unknown sample 4 had 11% of reads attributable to skeletal muscle biomarkers and 86% of reads attributable to adipose biomarkers. As a result of this pattern of biomarkers, the sample was correctly identified as adipose tissue. Unknown sample 5 had 100% of reads attributable to skeletal muscle biomarkers, and was correctly identified as a skeletal muscle sample. Unknown sample 6 had 100% of reads attributable to liver biomarkers, and was correctly identified as a liver sample. Interestingly, the liver sample used for unknown 6 was a fetal liver sample, indicating the ability of the assay to identify both fetal and adult liver samples.

## 4. Discussion

An MPS-based molecular organ tissue assay that can definitively identify internal organ tissue and could be used by any laboratory with forensic MPS capabilities will facilitate the investigation and prosecution of cases in which such potentially important contextual information about the organ tissue source of the DNA is present on a person, weapon, or location. Many of the cases impacted by such an assay include shootings or stabbings whereby the bullet or knife trajectory through the body, or the mere presence of particular internal organ tissue indicating proximity to, or involvement in, a significant trauma-producing event, might be demonstrated. The relative ease-of-use of such an assay by forensic molecular biologists will in time, once labs are up and running with MPS technology, ‘democratize’ the ability to routinely identify organ tissue when necessary instead of having to rely on specialized, and not always readily available, cellular pathology services.

We have made progress towards the development of such an assay with the prototype 46-plex MPS organ tissue ID system tested and evaluated in the present work. Further optimization, testing, and evaluation of the assay is necessary before it is ready for use in actual casework. This would include identifying suitably robust and highly expressed biomarkers (1) for other tissues such as spleen and spinal cord (to differentiate it from brain if possible), (2) with more specificity for stomach, adipose tissue, and trachea, and (3) to enable the identification of different regions of the brain. It will also be useful to specifically target and interrogate parts of the tissue-specific transcripts that possess coding region SNPs to help genetically identify the donor of the tissue, especially in situations involving mixtures of tissues from different individuals. The present work was carried out with extracted RNA from organ tissue samples obtained from commercial sources. The assay is currently in the process of being tested with total RNA isolated from in house *bona fide* tissue samples, including autopsied tissues. Subsequently, we will test the assay’s performance with mock casework samples in which organ tissue will be deposited on a variety of substrates and allowed to desiccate before analysis.

Categorical inference for the presence of a particular organ tissue was carried out here using a simple graphic method and/or agglomerative hierarchical clustering [42]. However, the final assay will likely incorporate a more formal probabilistic approach using partial least squares and linear discriminant analysis (PLS-DA) in order to determine the posterior probabilities for each of the possible tissues [43].

It is possible that in the future, the organ tissue targets will be incorporated together with body fluid-specific biomarkers into a combined comprehensive tissue identification assay to identify both externally secreted body fluids and internal organ tissues. Such an assay would require approximately 100 or fewer targets, a number that is easily accomplished with current multiplex MPS technology. This combined assay would simply become another modular component of the forensic scientist’s MPS armamentarium to be employed whenever necessary, along with DNA typing. Commercial vendors are already making plans to incorporate RNA-based body fluid identification into their MPS products, and could be easily expanded to include organ tissue markers.

## Figures and Tables

**Table 1:** Biomarker Composition of the 46-plex Targeted RNA Massively Parallel Sequencing (MPS) Multiplex for Human Organ Tissue Identification.

Tissue	Gene Name	Chromosome	Transcript ID	Illumina Assay ID
**Brain**	**SNAP25**	20	NM_130811	6650651
	**RTN1**	14	NM_021136	6597471
	**GABRA1**	5	NM_001127643	6769405
	**OPALIN**	10	NM_001040103	6690750
	**GFAP**	17	NM_002055	6760207
	**NEUROD6**	7	NM_022728	6608149
**Lung**	**SFTPB**	2	NM_198843	6822231
	**SFTPD**	10	NM_003019	6635044
	**SFTPA1**	10	NM_005411	6736962
**Trachea**	**BPIFB1**	20	NM_033197	6804173
**Liver**	**AMBP**	9	NM_001633	6846165
	**F2**	11	NM_000506	6834705
	**SPP2**	2	NM_006944	6646626
	**CFHR2**	1	NM_005666	6824671
	**F9**	X	NM_000133	6813125
	**MBL2**	10	NM_000242	6748563
	**AHSG**	3	NM_001622	6842654
	**C9**	5	NM_001737	6711440
**Skeletal**	**TNNI2**	11	NM_003282	6650981
**Muscle**	**MYLK2**	20	NM_033118	6800284
	**ATP2A1**	16	NM_004320	6782675
	**MYH2**	17	NM_017534	6700111
	**NEB**	2	NM_001164508	6690232
	**MYLPF**	16	NM_013292	6688633
**Heart Muscle**	**ITGB1BP3**	19	NM_170678	6650498
**Heart**	**MYBPC3**	11	NM_000256	6685046
	**NPPB**	1	NM_002521	6847931
	**NPPA**	1	NM_006172	6634864
	**TNNI3**	19	NM_000363	6715646
**Kidney**	**UMOD**	16	NM_003361	6842087
	**SLC12A1**	15	NM_001184832	6692344
	**SLC34A1**	5	NM_003052	6850242
	**SLC22A12**	11	NM_153378	6678522
**Adipose**	**TUSC5**	17	NM_172367	6779317
	**ADIPOQ**	3	NM_001177800	6795292
	**PLIN1**	15	NM_002666	6654705
**Intestine**	**FABP6**	5	NM_001130958	6641583
	**LCT**	2	NM_002299	6648509
	**CCL25**	19	NM_005624	6726865
	**DEFA5**	8	NM_021010	6669611
	**DEFA6**	8	NM_001926	6625127
**Stomach**	**PGA5**	11	NM_014224	6775995
	**PGA3**	11	NM_001079807	6973516
	**PGA4**	11	NM_001079808	6983051
	**GIF**	11	NM_005142	6675517
	**GKN1**	2	NM_019617	6798784

**Table 2:** Tissue Specificity of 46 Gene Candidates.

		Brain	Lung	Trachea	Liver	Sk.Mus	Heart	Kidney	Adipose	Sm.Int	Stomach
	N	5	3	3	4	4	4	3	2	4	3
	Avg Total	342,617	353,031	210,086	331,286	415,965	383,774	118,472	104,731	324,757	515,896
**BRN**	**SNAP25**	**167,707**									
	**RTN1**	**83,980**	*	1927					2045		
	**GABRA1**	**14,584**									
	**OPALIN**	**9698**									
	**GFAP**	**53,931**									
	**NEUROD6**	**9872_(4)_**									
**LUN**	**SFTPB**		**145,365**	*							
	**SFTPD**		**56,487**								
	**STFPA1**		**142,360**	*							
**TRA**	**BPIFB1**		11,338_(2)_	**190,738**							
**LIV**	**AMBP**	*			**165,375**				*		
	**F2**				**13,915**						
	**SPP2**				**6787_(3)_**						
	**CFHR2**				**21,586**						
	**F9**				**9090**						
	**MBL2**				**4649_(3)_**				*		
	**AHSG**	*			**93,723**						
	**C9**				**19,021**						
**SKM**	**TNNI2**			*		**106,756**			*		
	**MYLK2**					**19,082**					
	**ATP2A1**					**53,200**			*		
	**MYH2**			1586_(2)_		**43,511**			*		
	**NEB**			2491_(2)_		**67,115**			*		
	**MYLPF**			1583_(2)_		**125,702**			*		
**HRT**	**ITGB1BP3**					*	**20,005**				
	**MYBPC3**						**17,803**				
	**NPPB**						**14,539**				
	**NPPA**			*			**162,719**				
	**TNNI3**			*			**168,146**				
**KID**	**UMOD**							**53,914**			
	**SLC12A1**							**39,341**			
	**SLC34A1**							**16,085**			
	**SLC22A12**							**9133**			
**ADI**	**TUSC5**								**6842**		
	**ADIPOQ**								**11,854**		
	**PLIN1**			2533			*		**21,176**		
**INT**	**FABP6**									**36,487_(3)_**	
	**LCT**									*	
	**CCL25**									**4333_(3)_**	
	**DEFA5**									**165,872**	
	**DEFA6**									**114,731**	
**STM**	**PGA5**								*	*	**23,954**
	**PGA3**								*	*	**103,475**
	**PGA4**								*	*	**155,582**
	**GIF**										**7311_(2)_**
	**GKN1**										**18,557_(2)_**

Average (avg) read counts of each biomarker in tissue samples (calculated from N donors). For each tissue set, the avg total read counts (avg total) are listed. Numbers in parentheses represent the number of samples in which the biomarker was detected (provided only for biomarkers that were not detected in all samples). Average counts below 1000 were not considered significant and are not shown. * = expression observed in only one sample (avg value not possible). Shading: dark grey ≥10,000 read counts; light grey 5001–9999 read counts; no color ≤5000 read counts. Brain (BRN), lung (LUN), trachea (TRA), liver (LIV), skeletal muscle (SKM or Sk.Mus), heart (HRT), kidney (KID), adipose (ADI), small intestine (SMINT or Sm.Int), intestine (INT), stomach (STM).

**Table 3:** Contribution of Tissue Biomarker Classes to Expression Profiles.

	Brain	Lung	Trachea	Liver	Sk.Mus	Heart	Kidney	Adipose	Sm.Int	Stomach
Biomarkers	*N = 5*	*N = 3*	*N = 3*	*N = 4*	*N = 4*	*N = 4*	*N = 3*	*N = 2*	*N = 4*	*N = 3*
BRN	**99_(96-99)_**	0_(0-1)_	1	0	0	0	0	2_(1-3)_	0	0
LUN	0	**98_(94-100)_**	1_(0-2)_	0	0	0	0	0	0	0
TRA	0	2_(1-5)_	**89_(79-98)_**	0	0	0	0	0	0	0
LIV	1_(0-4)_	0	0	**100**	0	0	0	5_(5-10)_	0	0
SKM	0	0	4_(0-8)_	0	**100_(99-100)_**	0	0	**19_(0-38)_**	0	0
HRM	0	0	4_(0-13)_	0	0	**5_(2-10)_**	0	0	0	0
HRT	0	0	0	0	0	**95_(90-98)_**	0	0	0	0
KID	0	0	0	0	0	0	**100**	0	0	0
ADI	0	0	1	0	0	0_(0-1)_	0	**42_(25-59)_**	0	0
INT	0	0	0	0	0	0	0	0	**98_(92-100)_**	0
STM	0	0	0	0	0	0	0	**32_(0-63)_**	2_(0-8)_	**100**

Average percent contributions (**bold text**) and range of percent contributions (subscript text in parentheses, if applicable) of each tissue biomarker class is shown for each tissue (Brain (BRN), biomarker class comprised of six different gene markers; lung (LUN), biomarker class comprised of three different gene markers; trachea (TRA), biomarker class comprised of one gene marker; liver (LIV), biomarker class comprised of eight different gene markers; skeletal Muscle (SKM), biomarker class comprised of six different gene markers; heart muscle (HRM), biomarker class comprised of one gene marker; heart (HRT), biomarker class comprised of four gene markers; kidney (KID), biomarker class comprised of four different gene markers; adipose (ADI), biomarker class comprised of three different gene markers; intestine (INT), biomarker class comprised of five different gene markers; stomach (STM), biomarker class comprised of five different gene markers). Skeletal Muscle (Sk.Mus), small Intestine (Sm.Int). The number of donors (N) used to determine the averages are provided.

**Table 4:** Biomarker Sensitivity.

**Tissue**	**Input (ng)**	**Total Reads**	**% Cont**	**SNAP25**	**RTN1**	**GABRA1**	**OPALIN**	**GFAP**	**NEUROD6**		
**Brain**	**25**	253,551	99	120,086	71,499	8192	8698	36,150	6901		
	**10**	68,930	100	31,986	19,041	3036	2929	11,055	883		
	**5**	12,021	100	7256	3315			1450			
**Tissue**	**Input (ng)**	**Total Reads**	**% Cont**	**SFTPB**	**SFTPD**	**SFTPA1**					
**Lung**	**25**	641,600	100	232,508	31,220	377,872					
	**10**	103,030	100	43,874	6451	52,705					
	**5**	15,448	100	7612	808	7028					
**Tissue**	**Input (ng)**	**Total Reads**	**% Cont**	**BPIFB1**							
**Trachea**	**25**	75,524	92	69,521							
	**10**	63,490	99	62,901							
	**5**	Below MTR	--								
**Tissue**	**Input (ng)**	**Total Reads**	**% Cont**	**AMBP**	**F2**	**SPP2**	**CFHR2**	**F9**	**MBL2**	**AHSG**	**C9**
**Liver**	**25**	169,000	100	90.462	7565	3739	11,117	4328	1541	43,900	6348
	**10**	47,366	100	24,898	2598	695	3197	870	696	11,850	2562
	**5**	26,444	100	15,739	539	--	1807	592	--	6678	1089
**Tissue**	**Input (ng)**	**Total Reads**	**% Cont**	**TNNI2**	**MYLK2**	**ATP2A1**	**MYH2**	**NEB**	**MYLPF**		
**Sk. Mus**	**25**	234,740	100	50,132	9910	27,752	25,867	27,058	94,021		
	**10**	7053	100	706	--	942	1031	798	3576		
	**5**	20,865	100	3615	768	2061	2276	2190	9955		
**Tissue**	**Input (ng)**	**Total Reads**	**% Cont**	**ITGB1BP3**	**MYBPC3**	**NPPB**	**NPPA**	**TNNI3**			
**Heart**	**25**	550,182	100	46,661	30,405	29,711	149,253	294,152			
	**10**	33,336	100	2427	2115	1790	8145	18,859			
	**5**	17,489	100	765	832	893	5445	9554			
**Tissue**	**Input (ng)**	**Total Reads**	**% Cont**	**UMOD**	**SLC12A1**	**SLC34A1**	**SLC22A12**				
**Kidney**	**25**	30,360	100	9124	9356	7107	4773				
	**10**	14,360	100	4598	4556	3269	1937				
	**5**	Below MTR	--								
**Tissue**	**Input (ng)**	**Total Reads**	**% Cont**	**FABP6**	**LCT**	**CCL25**	**DEFA5**	**DEFA6**			
**Sm. Int**	**25**	1,227,602	100	189,948	--	--	690,364	347,290			
	**10**	557,746	100	74,829	--	--	314,865	167,268			
	**5**	398,704	100	55,869	--	--	223,287	119,549			
**Tissue**	**Input (ng)**	**Total Reads**	**% Cont**	**PGA5**	**PGA3**	**PGA4**	**GIF**	**GKN1**			
**Stomach**	**25**	486,450	100	227,634	49,505	178,348	8322	22,641			
	**10**	147,139	100	73,155	16,109	46,184	2379	9312			
	**5**	121,928	100	66,997	8539	36,550	2241	7601			

*% Cont = percent contribution*.

**Table 5:** Tissue Identification in a Six-Sample Blind Study Using the 46-plex Targeted RNA Sequencing Multiplex.

	Unk 1	Unk 2	Unk 3	Unk 4	Unk 5	Unk 6
**% Contr.**						
**BRN**	0	**100**	-	2	0	0
**LUN**	0	0	-	0	0	0
**TRA**	0	0	-	0	0	0
**LIV**	0	0	-	0	0	**100**
**SKM**	0	0	-	**11**	**100**	0
**HRM**	0	0	-	0	0	0
**HRT**	0	0	-	0	0	0
**KID**	0	0	-	0	0	0
**ADI**	0	0	-	**86**	0	0
**INT**	0	0	-	0	0	0
**STM**	**100**	0	-	1	0	0
**Analyst Conclusion**	**Stomach**	**Brain**	**No tissue detected**	**Adipose**	**Skeletal Muscle**	**Liver**
**Actual**	**Stomach**	**Brain (poly A)**	**Blank (water)**	**Adipose**	**Skeletal Muscle**	**Liver (fetal)**

Unk = unknown.

## Supplementary Materials

The following are available online at www.mdpi.com/2073-4425/8/11/319/s1, Table S1: List of Additional Biomarkers Tested and Rejected, Table S2: Specificity Amongst Body Fluids and Other Tissues. Figure S1: Intra-Class Biomarker Expression Differences within Target Tissues, Figure S2: Reproducibility.
