# Supplementary material for: Human Organ Tissue Identification by Targeted RNA Deep Sequencing to Aid the Investigation of Traumatic Injury
Source: Genes (Basel). 2017 Nov 10;8(11):319. doi: 10.3390/genes8110319 (PMC5704232; doi:10.3390/genes8110319)
Supplement: Supplementary file 1 [file genes-08-00319-s001.pdf]

**Figure S1.** Intra-Class Biomarker Expression Differences Within Target Tissues. The percent contribution of each individual biomarker was calculated (reads per biomarker/total reads per sample). The percentages from each biomarker within brain (A) and skeletal muscle (B) class are shown.

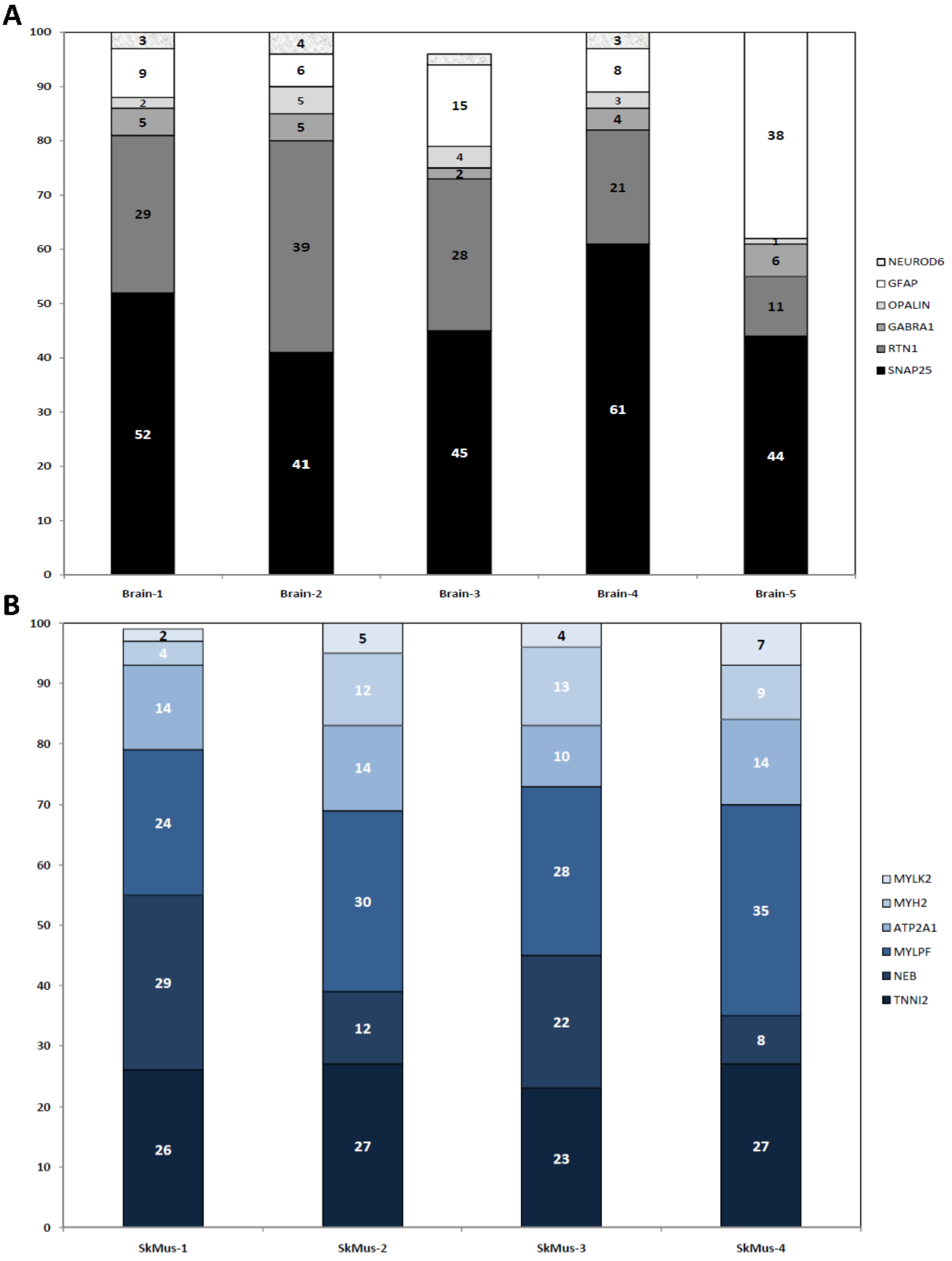

**Figure S2.** Reproducibility. Individual tissue samples were run in duplicate (-1, -2) using the same amount of input total RNA (50 ng). The percent contribution for individual biomarkers was calculated (reads per biomarker/total reads per sample). The percentages from each group of tissue specific biomarkers were combined in order to determine the percentage of reads per sample attributable to each tissue class. Percent reads attributable to each biomarker class are listed and represented by color. Y-axis – percent contribution; X-axis – tissue samples.

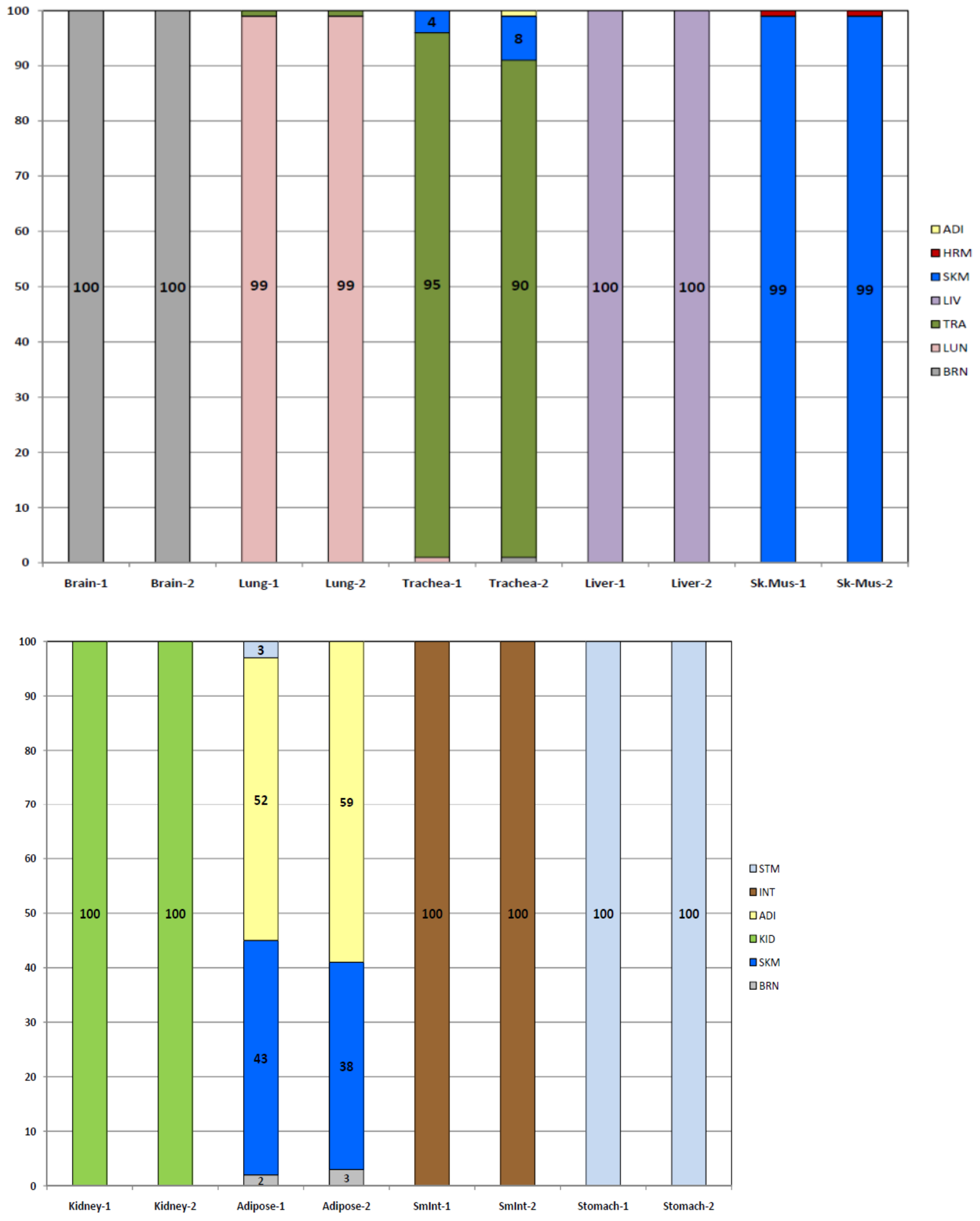

**Table S1.** List of Additional Biomarkers Tested and Rejected (Not Included in 46-plex Assay)

| Tissue | Gene Name | Illumina Assay ID | Reason for Rejection |
|---|---|---|---|
| Brain | OLIG2 | 6711104 | Low expression |
| | ZDHHC22 | 6601141 | Low expression |
| | CADP5 | 6649932 | Present in heart, adipose; low expression |
| Lung | SCGB1A1 | 6639464 | Present in adipose, trachea |
| | SCGB3A2 | 6696562 | Present in trachea |
| | ROS1 | 6844773 | Present in brain |
| | MS4A15 | 6688001 | |
| | RTKN2 | 6629508 | |
| Liver | VTN | 6717842 | Present in heart, brain; low expression |
| Sk. Mus | MYBPC2 | 6645240 | Present in adipose; lower expression than other candidates |
| | IDI2 | 6831880 | Present in adipose, lower expression than other candidates |
| | MYH1 | 6765512 | Present in adipose, lower expression than other candidates |
| | TNNT3 | 6597630 | Present in heart |
| | TNNI1 | 6776678 | Present in heart |
| | KCNA7 | 6974051 | Present in brain and vaginal secretions, very low expression |
| | MYH4 | 6808145 | Low expression |
| | LBX1 | 6764947 | Low expression |
| Heart | BMP10 | 6836630 | Present in liver |
| | MYL7 | 6855735 | Present in other tissues (bladder) |
| Kidney | SLC22A8 | 6815585 | Present in brain |
| | TMEM174 | 6664457 | Low expression |
| | MCCD1 | 6974627 | Present in vaginal secretions, low expression |
| | FXYD2 | | Not tested since suitable assay not available |
| Adipose | CIDEA | 6751245 | Present in heart, skin |
| | GYG2 | 6637210 | Low expression in several tissues |
| | WT1 | 6849763 | Present in heart, kidney, stomach |
| | LIPE | 6813131 | Present in brain, muscle, heart, trachea |
| Intestine | CPO | 6603314 | Present in brain, stomach |
| | KCNJ13 | 6699108 | Present in brain, liver, muscle, adipose; low expression |
| | MS4A10 | 6828362 | Present in small intestine, kidney |
| | SLC17A8 | 6661188 | Low expression; not detected in most small intestine samples |

**Table S2.** Specificity Amongst Body Fluids and Other Tissues**.** Average read counts of each biomarker in body fluid and tissue samples (calculated from N donors; only samples above MTR threshold were included in calculations) are listed. Numbers in parentheses represent the number of samples in which the biomarker was detected. No number in parentheses indicates that the biomarker was detected in all samples tested for that tissue. Shading: dark grey > 10,000 read counts; light grey 5,001 – 9,999 read counts; no color < 5,000 read counts. Average counts below 1,000 were not considered significant and are not shown.

| | Blood | Semen | Saliva | Vaginal | Menstrual | Colon | Spinal Cord |
|---|---|---|---|---|---|---|---|
| **N** | **5** | **4** | **4** | **4** | **4** | **1** | **1** |
| **Below MTR** | **4** | **4** | **4** | **4** | **4** | **0** | **0** |
| **SNAP25** | **16,743(1)** | -- | -- | -- | -- | | **56,086** |
| **RTN1** | **5,723(1)** | -- | -- | -- | -- | | **24,277** |
| **GABRA1** | | -- | -- | -- | -- | | |
| **OPALIN** | | -- | -- | -- | -- | | *2,894* |
| **GFAP** | *1,035(1)* | -- | -- | -- | -- | | **416,258** |
| **NEUROD6** | | -- | -- | -- | -- | | |
| **SFTPB** | | -- | -- | -- | -- | | |
| **SFTPD** | | -- | -- | -- | -- | | |
| **STFPA1** | | -- | -- | -- | -- | | |
| **BPIFB1** | | -- | -- | -- | -- | | |
| **AMBP** | | -- | -- | -- | -- | | |
| **F2** | | -- | -- | -- | -- | | |
| **SPP2** | | -- | -- | -- | -- | | |
| **CFHR2** | | -- | -- | -- | -- | | |
| **F9** | | -- | -- | -- | -- | | |
| **MBL2** | | -- | -- | -- | -- | | |
| **AHSG** | | -- | -- | -- | -- | | |
| **C9** | | -- | -- | -- | -- | | |
| **TNNI2** | | -- | -- | -- | -- | | |
| **MYLK2** | | -- | -- | -- | -- | | |
| **ATP2A1** | | -- | -- | -- | -- | | |
| **MYH2** | | -- | -- | -- | -- | | |
| **NEB** | | -- | -- | -- | -- | | |
| **MYLPF** | | -- | -- | -- | -- | | |
| **ITGB1BP3** | | -- | -- | -- | -- | | |
| **MYBPC3** | | -- | -- | -- | -- | | |
| **NPPB** | | -- | -- | -- | -- | | |
| **NPPA** | | -- | -- | -- | -- | | |
| **TNNI3** | | -- | -- | -- | -- | | |
| **UMOD** | | -- | -- | -- | -- | | |
| **SLC12A1** | | -- | -- | -- | -- | | |
| **SLC34A1** | | -- | -- | -- | -- | | |
| **SLC22A12** | | -- | -- | -- | -- | | |
| **TUSC5** | | -- | -- | -- | -- | | |
| **ADIPOQ** | | -- | -- | -- | -- | | |
| **PLIN1** | | -- | -- | -- | -- | | |
| **FABP6** | | -- | -- | -- | -- | | |
| **LCT** | | -- | -- | -- | -- | **27,728** | |
| **CCL25** | | -- | -- | -- | -- | **6,169** | |
| **DEFA5** | | -- | -- | -- | -- | **163,378** | |
| **DEFA6** | | -- | -- | -- | -- | **155,578** | |
| **PGA5** | | -- | -- | -- | -- | | |
| **PGA3** | | -- | -- | -- | -- | | |
| **PGA4** | | -- | -- | -- | -- | | |
| **GIF** | | -- | -- | -- | -- | | |
| **GKN1** | | -- | -- | -- | -- | | |
